# POOR ADHERENCE TO DRUG TREATMENT IN CHILDREN AND ADOLESCENTS WITH AUTOIMMUNE RHEUMATIC DISEASES

**DOI:** 10.1590/1984-0462/;2019;37;2;00019

**Published:** 2019-06-19

**Authors:** Clovis Artur Silva

**Affiliations:** a Universidade de São Paulo, São Paulo, SP, Brazil.

I read with great interest the study reported by Mioto e Silva et al.^1^ The investigators developed a relevant self-administered tool to assess adherence to medical and non-medical treatment in pediatric autoimmune chronic rheumatic diseases (PARDs). A pilot study evaluated a Pediatric Rheumatology Adherence Questionnaire, applied to caregivers, in two instances: diagnosis (the first four months of disease) and after six months. The four most important PARDs was included. Poor global adherence, defined as adherence <95%, was observed in 7/33 (21%) patients, poor adherence to medical treatment in 8/33 (24%), and a trend to correlation between socioeconomic factors and poor adherence was evidenced.

There are many factors associated with non/poor adherence to drug treatment in children and adolescents with PARDs, particularly low socioeconomic status, psychological stress of parents/patients, family dysfunction, drug unavailability, insurance type and coverage, unwanted adverse events and concomitant use of more than three different types of drugs daily.^2^ ^,^ ^3^ ^,^ ^4^ ^,^ ^5^

In addition, non/poor adherence to drug treatment and appointments in PARDs are more relevant issues, particularly in the second decade of life.^3^ ^,^ ^4^ ^,^ ^5^ Indeed, adolescents have a set of biological, psychosocial and brain maturation developments, becoming more independent, with caregiver autonomy, peer connection, beginning of sexual and romantic relationships.^3^ ^,^ ^4^ These findings may be delayed, exacerbated or impaired in adolescents with PARDs, contributing to low adherence to the use of immunosuppressive and biologic agents.

A recent web-based survey study evaluated epidemiology and management practices about childhood-onset systemic lupus erythematosus, including reports of 170/288 (59%) Latin American Pediatric Rheumatologists from 16 countries. Non-adherence to medications was the most important issue described by 97% of respondents, in spite of high frequencies of availability of glucorticosteroid, antimalarials and immunosuppressive drugs (>80%).^5^

Therefore, assessing and preventing low adherence poses a great challenge in clinical practice. Direct and indirect methods may help measure poor treatment adherence and should be regularly evaluated: self-administered questionnaires, structured interviews with patients/parents, electronic monitoring devices, adherence history and measurement of serum/drug metabolite levels.^2^ ^,^ ^4^ Specific programs for this purpose should be developed to improve compliance. Behavior and education strategies about the disease and treatments, through constructive dialogue at individual/group level with multidisciplinary teams may help PARDs patients improve adherence.^2^ ^,^ ^4^
